# Muscle hypertrophy and neuroplasticity in the small bowel in short bowel syndrome

**DOI:** 10.1007/s00418-023-02214-4

**Published:** 2023-07-03

**Authors:** Rasul Khasanov, Daniel Svoboda, María Ángeles Tapia-Laliena, Martina Kohl, Silke Maas-Omlor, Cornelia Irene Hagl, Lucas M. Wessel, Karl-Herbert Schäfer

**Affiliations:** 1 Department of Pediatric Surgery, University Hospital Mannheim, Medical Faculty Mannheim of Heidelberg University, Theodor-Kutzer-Ufer 1-3, 68167 Mannheim, Germany; 2 Department of Pediatric and Adolescent Medicine, University Medical Center Schleswig-Holstein, Ratzeburger Allee 160, 23538 Lübeck, Germany; 3 Enteric Nervous System Group, University of Applied Sciences Kaiserslautern, Amerikastrasse 1, 66482 Zweibrücken, Germany; 4 Carl Remigius Medical School, Charles de Gaulle Str. 2, 81737 Munich, Germany

**Keywords:** Enteric neurons, ENS, Nestin, PGP 9.5, Short bowel syndrome, Bowel resection

## Abstract

Short bowel syndrome (SBS) is a severe, life-threatening condition and one of the leading causes of intestinal failure in children. Here we were interested in changes in muscle layers and especially in the myenteric plexus of the enteric nervous system (ENS) of the small bowel in the context of intestinal adaptation. Twelve rats underwent a massive resection of the small intestine to induce SBS. Sham laparotomy without small bowel transection was performed in 10 rats. Two weeks after surgery, the remaining jejunum and ileum were harvested and studied. Samples of human small bowel were obtained from patients who underwent resection of small bowel segments due to a medical indication. Morphological changes in the muscle layers and the expression of nestin, a marker for neuronal plasticity, were studied. Following SBS, muscle tissue increases significantly in both parts of the small bowel, i.e., jejunum and ileum. The leading pathophysiological mechanism of these changes is hypertrophy. Additionally, we observed an increased nestin expression in the myenteric plexus in the remaining bowel with SBS. Our human data also showed that in patients with SBS, the proportion of stem cells in the myenteric plexus had risen by more than twofold. Our findings suggest that the ENS is tightly connected to changes in intestinal muscle layers and is critically involved in the process of intestinal adaptation to SBS.

## Introduction

Short bowel syndrome (SBS) is a severe, life-threatening condition and one of the leading causes of intestinal failure in children; as a result of massive bowel resection or loss of resorptive area, the absorption of the remaining intestine is not sufficient, causing maldigestion and malabsorption (Goulet and Sauvat 2006; Oliveira et al. 2012). Accordingly, intestinal adaptation after extensive small bowel resection is crucial for enhancing the absorptive and digestive capacity. The functional and structural changes in intestinal adaptation comprise smooth muscle hyperplasia, enterocyte proliferation (O’Brien et al. 2001; Oliveira et al. 2012; Martin et al. 2008), increased villus height, crypt depth, and changes in binding proteins and receptors (Stephens et al. 2010; Stern et al. 2000; Vomhof-DeKrey et al. 2021; Sukhotnik et al. 2016). However, to date, most of the studies on SBS adaptation mainly focused on intestinal epithelium and the role of growth and angiogenetic factors (McMellen et al. 2010; Tappenden 2014a; Lin et al. 2019; Martin et al. 2009).

Usually many patients with SBS suffer from relevant pathological dilatation of the remaining small intestine, including the formation of reservoirs and stasis of intestinal contents. Clinically, these changes are manifested by increased malabsorption and maldigestion, excessive proliferation of bacterial microflora in the small intestine, which leads to complications such as bacterial overgrowth, d-lactate acidosis, translocation of intestinal bacteria into extraintestinal organs, and mucosal ulceration with the development of intestinal bleeding (Weih et al. 2012; Ives et al. 2016).

The basis for dilatation of the small intestine could be induced by changes in the muscle layers of the small intestine or alterations within the intrinsic nervous system of the gut, the enteric nervous system (ENS). Although there is no literature consensus about the role of the muscle layers in the small intestine adaptation after SBS (Chen et al. 2012, 2015, 2013; Martin et al. 2008), some studies have described that reduced ENS function enhances intestinal mucosal adaptation after massive small bowel resection (Garcia et al. 1999; Hitch et al. 2012). Also, the ENS participates in the glucagon-like peptide 2 (GLP-2) pathway, which is known to enhance intestinal adaptation and regulates several intestinal adaptive processes, including epithelial proliferation, apoptosis, and inflammation (Kaunitz and Akiba 2019; Fleming et al. 2020). Moreover the ENS adapts itself continuously to changing environmental conditions like diet (Fichter et al. 2011), mechanical bowel movements, inflammatory responses (Collins et al. 1992), and the microbiome (Grundmann et al. 2016), which strongly influence neuronal differentiation and plasticity. The ENS also regulates primary gut functions such as peristalsis, electrolyte secretion, and blood supply (Furness 2012). Furthermore, the ENS interacts with the intrinsic gut immune system (Wood 2004; Niesler et al. 2021).

Importantly, the ENS is crucial for bowel motility. For instance, in conditions like Hirschsprung’s disease, where parts of the ENS are absent or reduced, the affected part of the gut has no peristaltic or propulsive motility (Lotfollahzadeh et al. 2021). Here, the neuronal homeostasis seems to be maintained by new neurons formed from myenteric ganglia precursor cells. These neuronal precursors express both nestin and p75NTR, but not the pan-glial marker Sox10 (Kulkarni et al. 2017). Nestin acts as a neuronal precursor marker (Grundmann et al. 2016). Nestin is a well-described marker for neural stem cells and it is abundantly expressed during embryogenesis in the human embryonic gut (Rauch et al. 2006). In the adult tissue, nestin-expressing cells are mainly restricted to defined niches where they may function as a quiescent resource capable of proliferation, differentiation, and migration (Wiese et al. 2004; Park et al. 2010). After injury, nestin expression is upregulated in the central nervous system, skeletal muscle, and gastrointestinal tract (Vanderwinden et al. 2002; Rauch et al. 2006; Cantarero Carmona et al. 2011). Nestin-green fluorescent protein in enteric neurons in transgenic mouse model represents a marker for neuronal plasticity (Grundmann et al. 2016). Therefore, nestin is the appropriate indicator of changes in ENS after massive small bowel resection.

However, the direct influence of ENS on intestinal adaptation after massive small bowel resection has not been described yet. Since it is known that both the ENS and intestinal smooth muscles are tightly connected, we designed this study to analyze the role of both smooth muscle tissue and the ENS on the intestinal adaptation after massive small bowel resection in animal models.

## Materials and methods

### Animals and tissue collection

Animal experiments were conducted with the approval of the local ethic committee (Luebeck University, V 312-72241.122-24 (43-3/06)) on adult Wistar rats (260–360 g). Twelve rats underwent a massive resection of the small intestine to induce SBS (rSBS) as described by Lei et al. (2011). Rats were anesthetized with an intraperitoneal injection of ketamine (40 mg/kg) and Rompun (4 mg/kg). The small intestine was resected, leaving 10 cm jejunum and 5 cm ileum, so approximately 80% of the small intestine was resected. Finally, a marking suture was applied 5 cm orally to the anastomosis. After bowel resection in the rSBS group, end-to-end anastomoses with interrupted Vicryl 6/0 sutures were performed. Simultaneously, a sham laparotomy without small bowel transection was performed in 10 rats, and these animals were used as controls. In the control group, marking sutures on the small bowel 10 and 5 cm adjacent to the colon were applied. One rat out of 12 in the rSBS group died during the surgery. No antibiotics were administered. In general, rats with SBS lost 10–15% of their original body weight during the first week. By the end of the second week, most animals had regained their preoperative weight.

Two weeks after surgery, all animals were killed by cervical dislocation, and the remaining jejunum and ileum were harvested. The lumen was flushed with ice-cold phosphate-buffered saline before further processing. The specimens orally and anally from intestinal anastomosis were harvested and marked as jejunum and ileum samples, respectively. The samples were fixed in 4% formaldehyde overnight, embedded in paraffin, sectioned at 5 µm thickness, and processed for hematoxylin–eosin (HE), Picrosirius Red, and immunohistochemical stainings (smoth muscle actin and nestin).

### Immunohistochemical staining

HE staining was conducted according to the standard protocol with the Leica autostainer LX.

Serial paraffin-embedded sections of small bowel were stained with Picrosirius Red according to the manufacturer’s protocol, using a commercial kit (Nr. 13425 Morphisto, Frankfurt am Main, Germany). Briefly, paraffin-embedded sections were deparaffinized and rehydrated through a series of xylene and graded alcohol washes, followed by staining with Weigert iron hematoxylin for 15 min. Slides were rinsed in distilled water, washed for 8 min in running tap water, and again rinsed in distilled water. Afterwards slides were stained for 1 h in a Sirius Red solution. Next, the sections were treated with acetic acid 30% twice for 1 min. Slides were submersed in 96% ethanol twice for 4 min, quickly dehydrated in graded ethyl alcohols, cleared with xylene, and mounted with xylene-based mounting medium (Nr. 13425 Morphisto, Frankfurt am Main, Germany).

Smooth muscle actin staining started with standard deparaffinization and rehydration (70%, 80%, 90%, 2 × 100% ethanol, 3 × xylol; 30 s each) of the slides. Unmasking the antigens was performed in a steamer in Tris–EDTA buffer pH 9.0 (DAKO K8002); permeabilization was performed for 10 min with 0.5% Triton X100; blocking was performed with Normal Goat Serum (1:10; DAKO X0907) for 30 min. The incubation of mouse-anti-smooth muscle actin AF 594 nm (1:100; Santa Cruz: sc-53142) was for 1 h. Finally, samples were incubated with DAPI and mounted.

Nestin–peroxidase staining was performed with the ENVISION KIT (DAKO) as follows: unmasking with citrate buffer pH 6.0 (DAKO S2369) at 95 °C for 1 h; blocking with 5% BSA (Serva 11930) for 1 h. Incubation with mouse-anti-nestin (1:200; Chemicon Int) for 1 h. After incubation with 3% hydrogen peroxide, samples were incubated with DAKO EnVision + System-HRP labelled polymer anti-Mouse (DAKO K4000) as a secondary antibody and visualized with DAB Substrat-Chromogen Set (DAKO K3468). Then, samples were dehydrated (70%, 80%, 90%, 2 × 100% ethanol, 3 × xylol; 30 s each).

Nestin fluorescent staining was performed as follows: unmasking the antigens with citrate buffer pH 6.0 (DAKO S2369) at 95 °C for 1 h; blocking with Normal Goat Serum (1:10; DAKO X0907), for 1 h, incubation of mouse-anti-human nestin (1:200; Chemicon Int) for 1 h, and incubation with the secondary antibody Alexa Flour® goat-anti-mouse 488 (1:500) for 1 h. Finally, samples were stained with DAPI and mounted.

Potential non-specific binding of secondary antibodies was assessed by omission primary antibodies in sections that were otherwise treated in a similar manner. Also, the specificity of the primary antibodies was confirmed by the use of isotype controls.

We have previously worked using the antibodies included in the present study in many other research works, on formalin-fixed tissues from either stillborn or aborted human fetuses and from adults, as well as on cell cultures of rat and human enteric neurospheres (Rauch et al. 2006; Hagl et al. 2013a, 2013b; Schafer et al. 2003). The specificity of the Picrosirius Red staining has been tested on the human and rat tissues by Yu et al. (2017) and Lattouf et al. (2014). The specificity of Smooth Muscle Actin (B4) antibody (Santa Cruz Biotechnology, sc-53142) has been tested on rat and human tissues and cells by immunofluorescence analysis (Breikaa et al. 2013; Tian et al. 2015).

### Image analysis and measurements of animal samples

The diameter of the small bowel and thickness of the muscle layers were measured. The thickness of muscle layers was measured each 500 µm in the pre-anastomotic small intestine (jejunum) and the post-anastomotic intestine (ileum) in the rSBS group and control group. Both the bowel diameter and thickness of muscle layers impact the amount of muscle tissue in the bowel. Thus, the area of muscle circumference was calculated according to the following formula: area of muscle circumference (area of a ring) = π (*R*^2^ − *r*^2^), where* R* is the outer radius and* r* is the inner radius.

Nestin–peroxide-stained samples were subjected to morphometric analysis to analyze the quantity of nestin-stained myenteric ganglia and nestin-positive areas in muscle layers in a whole crosscut section (in bowel circumference).

We used nestin immunofluorescence staining to analyze nestin-positive areas within a single myenteric ganglion. The myenteric plexus was identified, and the ratio of nestin-positive area to the total area of myenteric plexus was calculated.

**Fig. 1:**
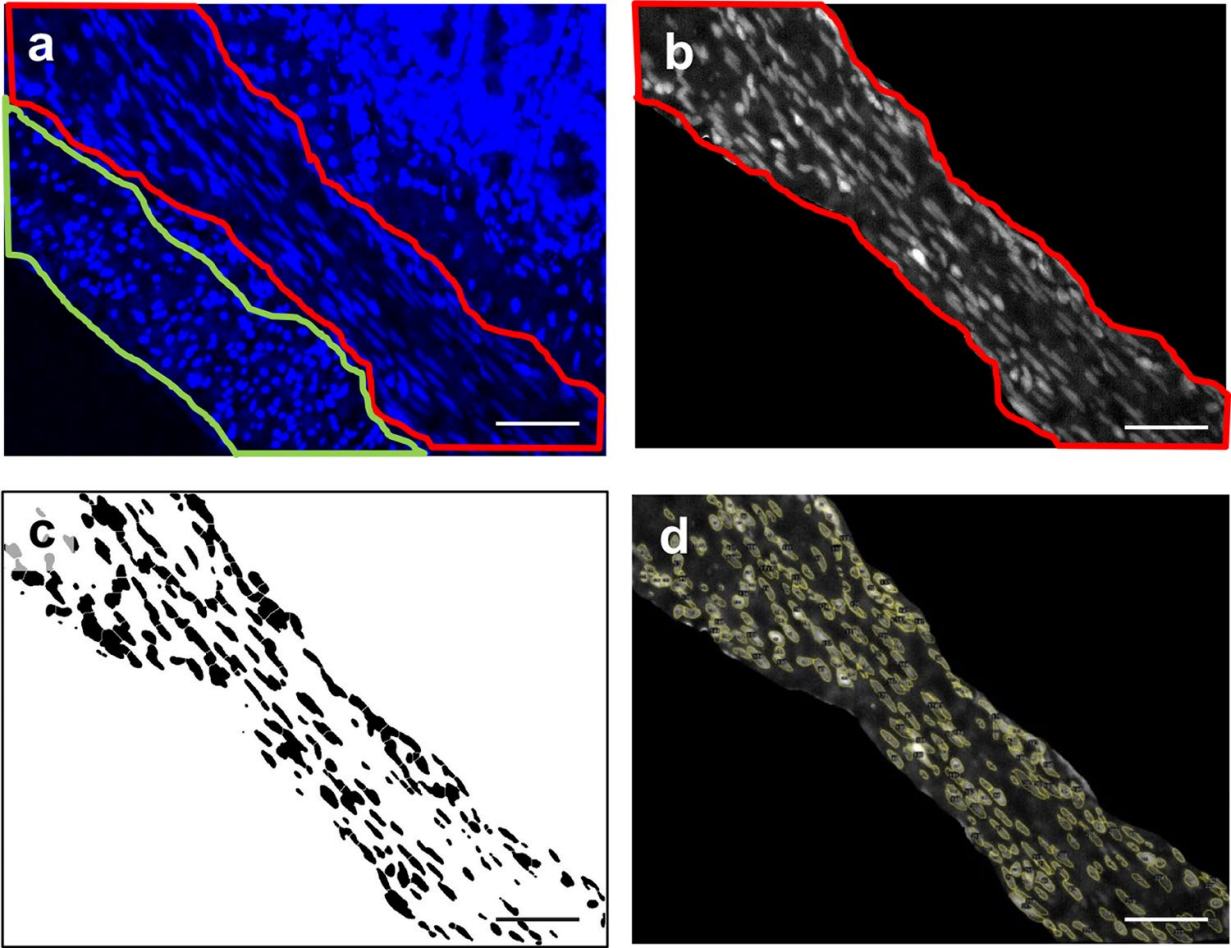
Process of nuclear analysis in the muscle layers of the small intestinal wall (rat). **a** DAPI staining of nuclei. Areas of longitudinal (green) and circular (red) muscle were outlined. **b** Isolated circular muscle layer in 16-bit picture using ImageJ software. **c** Mapping of the picture in** b** using ImageJ software based on binary imaging.** d** Counting and analyzing of the circular muscle layer using ImageJ software. Scale bar is 50 µm

Finally, to determine whether the changes in thickness of the muscle layers of the bowel were due to hypertrophy or hyperplasia, we analyzed the morphology, size, and number of nuclei in the different bowel muscle layers (circular and longitudinal) using the ImageJ software (Version 1.53a; NIH, USA). On the basis of the DAPI channel pictures, the areas of the circular (CML) and longitudinal muscle (LML) layers were selected and isolated. Then, the individual layers were separated, thresholded, binarized, the background was removed, despeckled, watershed, and finally the exact individual nuclei shapes were selected, measured, and analyzed with the software (Fig. 1). In total, for the control groups for the oral (proximal) and anal (distal) samples, at least 6 samples (and 5–6 pictures/sample) were quantified. For the SBS (KD) sections, at least 6 samples (and 3–6 pictures/sample) were analyzed for the oral (proximal) segments, while at least 5 samples (and 3–8 pictures/sample) were quantified for the anal (distal) part.

### Human samples

Human samples were collected according to the approval of the local ethic committee (2016-525N-MA) of the Medical Faculty Mannheim University of Heidelberg.

We conducted a comparative analysis of pathomorphological changes in minor resections of the small bowel, fulfillment of which was medically required during the surgical treatment of children with SBS (four patients), which made up the main group.

The control group was composed of patients who underwent resection of minor small bowel segments because of the medical indication, specifically Meckel’s diverticulum (*n* = 3) and foreign body (*n* = 1) causing intestinal obstruction with the need of minor resection. None of the patients in the control group had SBS or intestinal failure.

The patients were age-matched: the age ranged from 4 months to 7.5 years in patients with SBS and from 4 months to 10.5 years in the control group. The main and the control group had an identical gender composition: one boy and three girls.

### Staining and microscopy of human samples

After removal of the tissue from the patient, the specimens were transferred directly from surgery to the laboratory, fixed in 4% paraformaldehyde overnight, embedded in paraffin, and sectioned at 4 µm thickness. Then, samples were dehydrated (70%, 80%, 90%, 2 × 100% ethanol, 3 × Neo-Clear; 30 s each), incubated in the steamer with citrate buffer pH 6.0 (DCS CL009C-100) for 45 min, followed by an enzymatic unmasking with Protease K (DAKO Agilent S3020) for 8 min at 95 °C. Next, samples were permeabilized with 0.5% Triton X100, and blocked with Normal Alpaca Serum (Jackson ImmunoResearch Laboratories, Inc.) for 1 h and then incubated with the corresponding primary antibodies: mouse-anti-human nestin (1:100; Millipore MAB 5326) and rabbit-anti-PGP9.5 (1:250; DAKO Agilent Z5116) for 1 h at room temperature. The negative control was incubated with Tris-phosphate buffer + 0.5% TWEEN 20. After three washes in PBS-Tween, samples were incubated for 1 h with the corresponding secondary antibodies: Alexa Fluor® alpaca-anti-rabbit 594 nm (1:500), Alexa Fluor® alpaca-anti-mouse 488 nm (1:500) (Jackson ImmunoResearch Laboratories, Inc.). Regarding nuclear staining, slides were either shortly incubated with DAPI (4′,6-diamidino-2-phenylindole dihydrochloride) (Thermo Fisher 65-0880-92) and DAKO Fluorescence Mounting Media (DAKO Agilent S302380), or covered up directly with Roti®MOUNT Fluore Care DAPI (Carl Roth HP20.1).

### Image acquisition

Pictures were taken with the light and fluorescence inverted microscope Keyence BZ-9000E, (Keyence Corporation, Higashi-Nakajima, Higashi-Yodogawa-ku, Osaka, 533-8555 1, Japan) using the objectives ×20 (Nicon Plan Apo, 20X/0.75, DIC N2 OFN25 WD 1.0) and ×40 (Nicon Plan Apo, 40X/0.95, DIC M/N2 OFN25 WD 0.14). All pictures were acquired and processed with the Keyence camera and software. The camera is installed in the Keyence BZ-9000E microscope (2/3 inch, 1.5 million pixel monochromer CCD Fotosensor (with LC Filter)) with shooting condition 1360 × 1024 pixel. The acquisition software is delivered with the Keyence BZ-9000E microscope: Program “BZ-II Viewer” (Version: 2.1.00a0.0100.0101.0100.0003). Image acquisition was performed in the following conditions: detector gain +12 dB, bit depth 24, fluorescence filters OP-66834 BZ Filter DAPI-BP (excitation wavelength 360/40 nm, absorption wavelength 460/50 nm), OP-66836 BZ Filter GFP-BP (excitation wavelength 470/40 nm, cold mirror wavelength 505 nm, absorption wavelength 535/50 nm), OP-66838 BZ Filter TexasRed (excitation wavelength 560/40 nm, absorption wavelength 630/60 nm), excitation time 1/15–1/90 s; multifluorescence image acquisitions were performed successively. The obtained pictures were analyzed using the “BZ-II Analyser” (Version 2.1) software, which is delivered with the Keyence BZ-9000E microscope.

### Statistical analysis

Statistics were analyzed with JMP software. Data distribution was determined using the Shapiro–Wilk test and visual analysis. Nonparametric tests were used for data that did not satisfy the normal distribution. In addition to mean and standard deviation, the median and the 25th and 75th percentiles were calculated. Normally distributed data are presented as mean ± standard deviation, and non-normally distributed data are presented as median (25th percentile–75th percentile). Analysis and comparison for quantitative analysis were performed using the Student* t *test for normally distributed data and the Wilcoxon test for non-normally distributed data. For qualitative attributes, Fisher’s exact and chi-square tests were used. Statistical significance was accepted when the *p* value was 0.05 or less (*p < 0.05). Nonparametric correlations were performed with the Spearman’s correlation test.

## Results

### The muscle layers is thickened in SBS

In order to investigate the intestinal adaptation of the muscle–neuronal complex, we analyzed the smooth muscle morphology after small bowel resection. To do so thickness of longitudinal and circular muscle layers and length and diameter of the intestine were measured.

**Fig. 2:**
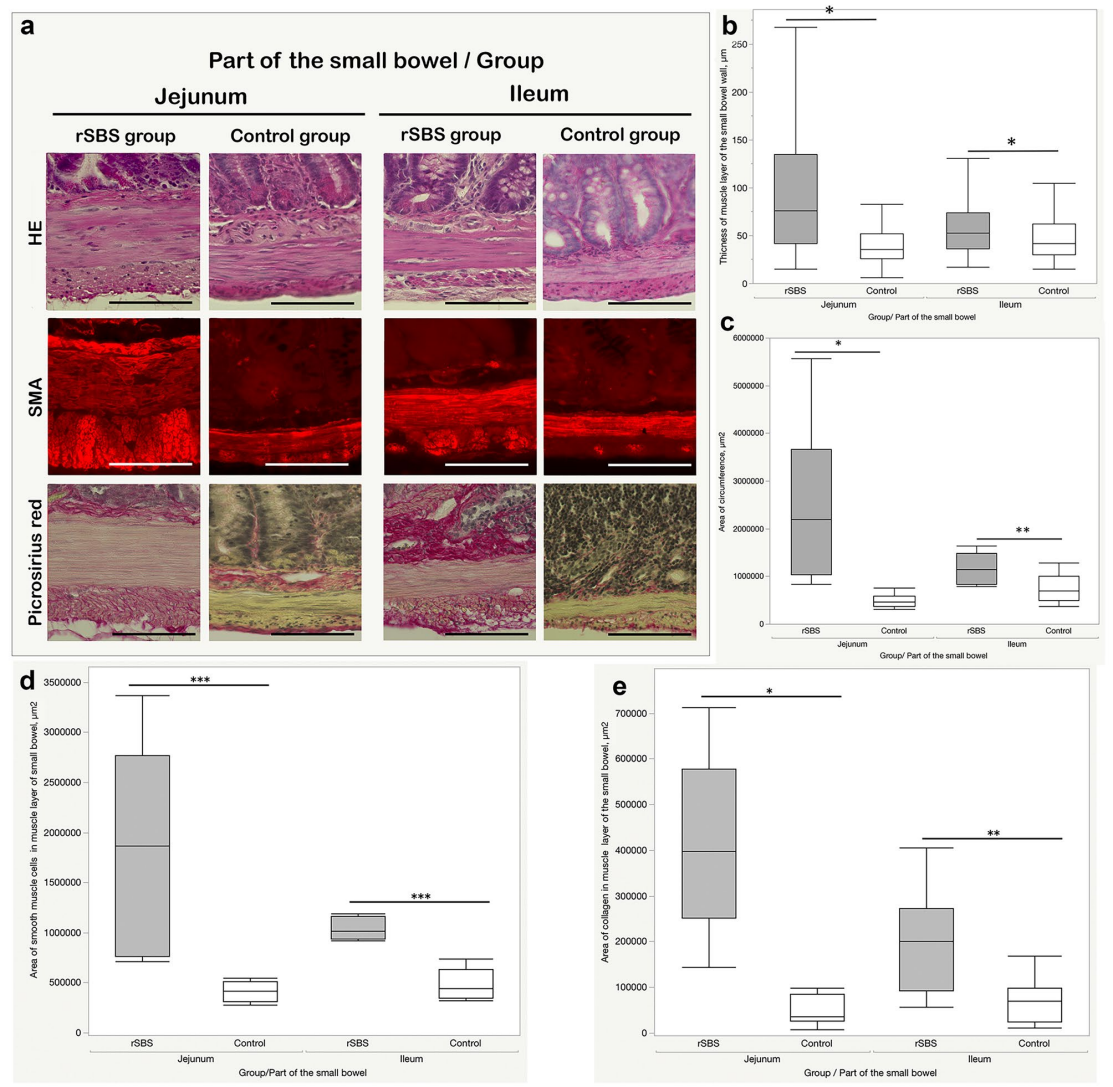
Comparison thickness of muscle layer of the small bowel between short bowel syndrome group (rSBS) and control group sections (rat): **a** Hematoxylin–eosin (HE), Picrosirius Red, and fluorescent smooth muscle actin (SMA) staining of jejunum and ileum; in Picrosirius Red staining, collagen fibers have a red color. In SMA staining, smooth muscle cells present a fluorescence red color, scale bar is 50 µm; **b** significant increase in the thickness of muscle layer in rSBS compared to control in jejunum and ileum; **c** area of muscle circumference of the small bowel (rat)—significant increase of the muscle circumference of the small bowel in rSBS compared to control in jejunum and ileum; **d** area of smooth muscle cells in muscle circumference of the small bowel (rat)—significant increase in rSBS compared to control in jejunum and ileum; **e** area of collagen in muscle circumference of the small bowel (rat)—significant increase in rSBS compared to control in jejunum and ileum. Line within box represents the median; the top and bottom of the box represent the 75th and 25th percentile, respectively. The whiskers indicate the maximum and minimum (not including outliers). Wilcoxon test: *p < 0.0001, **p = 0.0051, ***p = 0.0004

The rats with short bowel resection showed significant increase of the thickness of smooth muscle layer compared to controls (Fig. 2a). The thickness of the muscle layer of the small bowel significantly increased in the rSBS group in both jejunum (76 µm (42–135 µm)) and ileum (53 µm (36–74 µm)) compared to the control group in jejunum (35.3 µm (26.25–51.75 µm) *p* < 0.0001) and ileum (42 µm (30–62 µm) *p* < 0.0001) (Fig. 2b). Next, we measured the diameter of the small bowel. The rSBS group showed a significant increase in both jejunum (7573 µm (6428–8334 µm), *p* < 0.0001) and ileum (6020 µm (4837–6706 µm), *p* < 0.0012) in comparison to the control group (jejunum 4128 µm (377–4511 µm); ileum 4757 µm (4405–5134 µm)).

Increased muscle thickness and small bowel diameter in rSBS lead to an amplified quantity of muscle tissue in the intestinal muscle layers. The area of muscle tissue was measured by calculating the area of muscle circumference of the small bowel, as described above in the “Materials and methods”. The area of muscle circumference in the rSBS group was significantly increased in jejunum (2,194,184 µm^2^ (1,042,226–3,664,209 µm^2^), *p* < 0.0001) and ileum (1,144,426 µm^2^ (838,496.3–1,487,406 µm^2^), *p* = 0.0051) compared to the control (jejunum 463,732.4 µm^2^ (368,656.4–580,718.6 µm^2^), ileum 697,614.1 µm^2^ (497,905.1–1,012,602 µm^2^)) (Fig. 2c).

To determine which compartment or cell type contributes to increasing the thickness of the muscle layer in SBS, gut sections were stained with antibodies against smooth muscle actin to identify and quantify the amount of smooth muscle cells. Picrosirius Red staining identified the connective tissue (collagen I and III).

The analyses showed that the area of muscle circumference in the rSBS group increases due to significant amplification of smooth muscle cells in both jejunum (1,866,439 µm^2^ (758,937.2–2,772,582 µm^2^)) and ileum (1,013,129 µm^2^ (937,420.6–1,158,178 µm^2^)) compared to the control group (jejunum 418,598.6 µm^2^ (310,687.2–515,031.9 µm^2^) *p* = 0.0004); ileum 440,803.4 µm^2^ (342,159.7–628,886.6 µm^2^) *p* = 0.0004) (Fig. 2d). The was also a significant amplification of collagen in the area of muscle circumference in the rSBS group in jejunum (398,325.5 µm^2^ (251,290.8–577,731.8 µm^2^), *p* < 0.0001) and the ileum (200,106.4 µm^2^ (92,736.8–273,180 µm^2^), *p* = 0.0051) compared to the control (jejunum 35,445.03 µm^2^ (27,163.9–85,353.1 µm^2^); ileum 69,707.2 µm^2^ (24,968.4–99,235.6 µm^2^) (Fig. 2e).

**Fig. 3:**
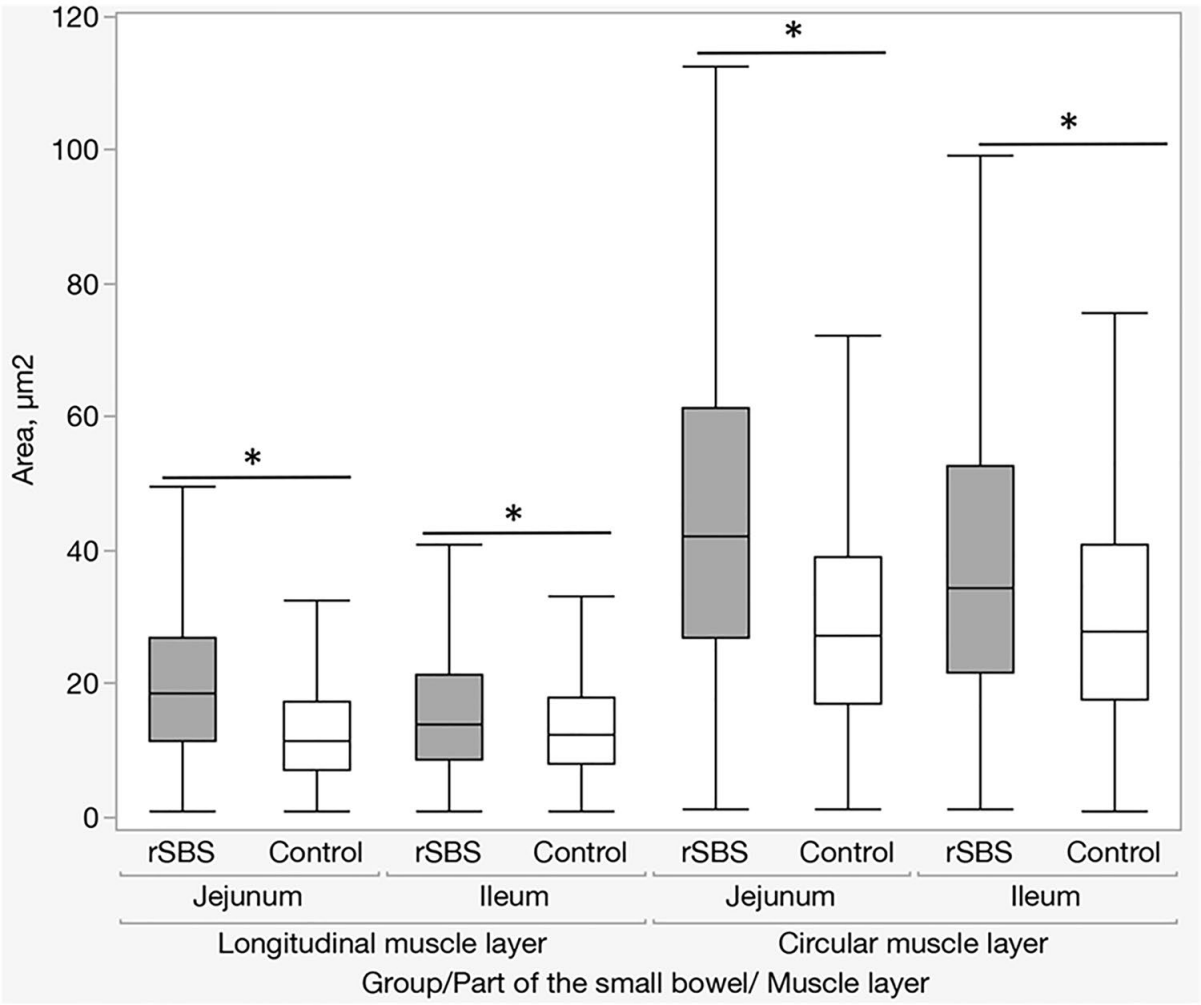
Size of the nuclei (area) (rat). Significant increase of the size of the nuclei in rSBS compared to control in jejunum and ileum. Line within box represents the median; the top and bottom of the box represent the 75th and 25th percentile, respectively. The whiskers indicate the maximum and minimum (not including outliers). Wilcoxon test: *p < 0.0001

The next aspect of our study was to evaluate whether the increase in the volume of muscle tissue of the small intestine in SBS is the result of hypertrophy or hyperplasia of muscle tissue. For this, we carried out a morphometric analysis of nuclei (size and density) of muscle cells of the bowel wall. The analysis showed that in SBS, the size of the nuclei (area) is significantly increased in comparison with the control group in both the longitudinal and circular muscle layers, and in the jejunum and the ileum (Table 1, Fig. 3).

**Table 1:** Size of the nuclei (the area of the nucleus in the cross section) (rat)

Part of the bowel	Muscle layer	Group	Minimum	25th percentile	Median	75th percentile	Maximum	*P* Wilcoxon
Ileum	Circular	Control	1.012	17.578	27.966	40.88275	255.67	< 0.0001
		rSBS	1.084	21.607	34.289	52.752	196.27	
	Longitudinal	Control	1.012	7.949	12.429	18.066	103.99	< 0.0001
		rSBS	1.012	8.527	13.947	21.462	117.79	
Jejunum	Circular	Control	1.156	17.126	27.388	39.18425	117.79	< 0.0001
		rSBS	1.156	26.954	42.057	61.279	178.85	
	Longitudinal	Control	1.012	7.082	11.345	17.343	73.925	< 0.0001
		rSBS	1.012	11.49	18.572	26.882	89.1

**Fig. 4:**
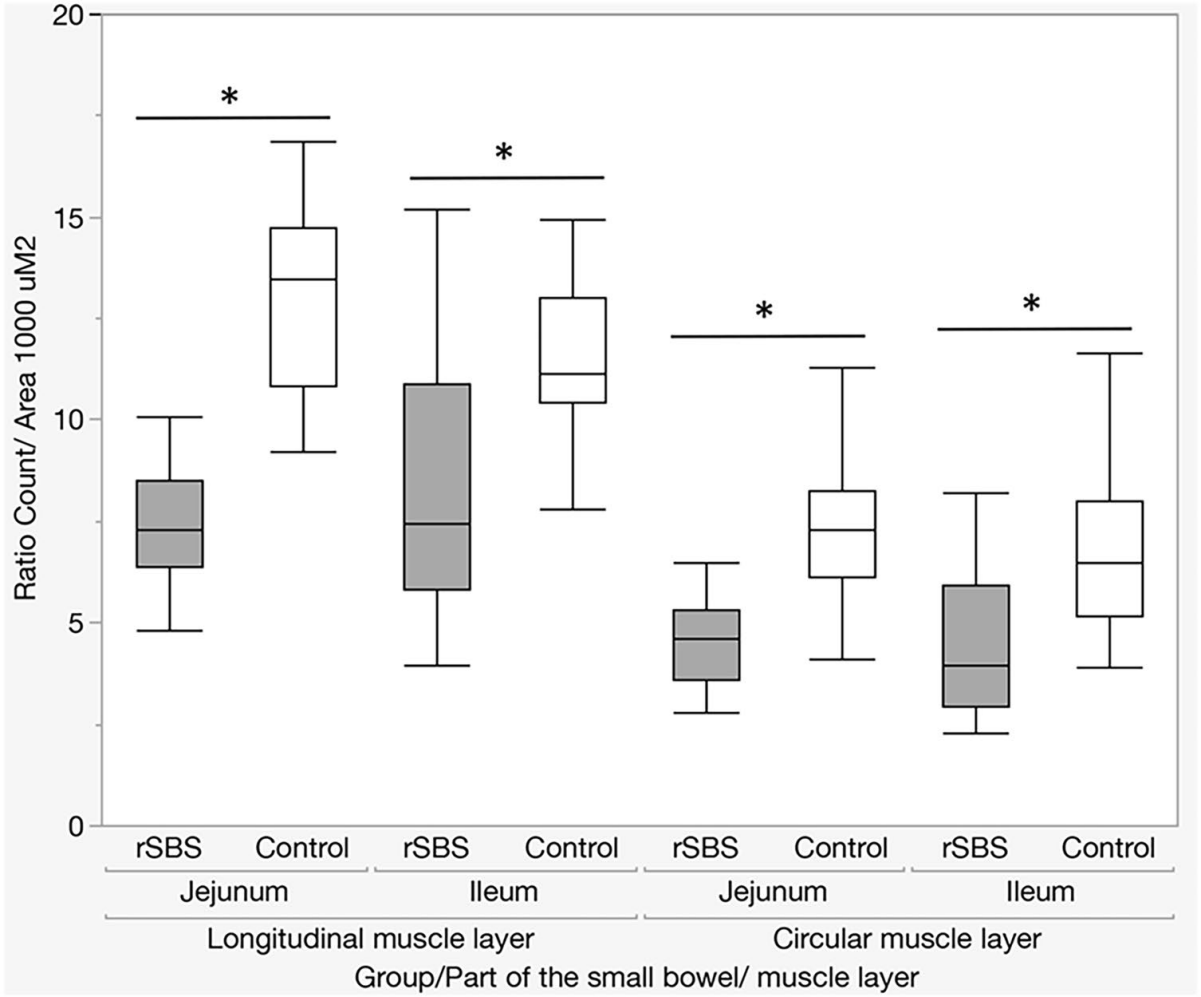
Number of nuclei in a 1000 μm^2^ area of muscle layer (rat). The was a significant increase in the number of nuclei in the muscle layer in rSBS compared to control in jejunum and ileum. Line within box represents the median; the top and bottom of the box represent the 75th and 25th percentile, respectively. The whiskers indicate the maximum and minimum (not including outliers). Wilcoxon test: *p < 0.0001

However, the number of nuclei in a 1000 μm^2^ area by SBS is significantly smaller compared with the control group in both the longitudinal and transverse muscle layers, and the jejunum (before the anastomosis) and the ileum (after anastomosis) (Table 2, Fig. 4).

**Table 2:** Number of nuclei in a 1000 μm^2^ area of muscle layer (rat)

Part of the bowel	Muscle layer	Group	Minimum	25th percentile	Median	75th percentile	Maximum	*P* Wilcoxon
Ileum	Circular	Control	0	4.278	5.153	6.489	8.012	< 0.0001
		rSBS	2.300	2.625	2.959	3.970	5.906	
	Longitudinal	Control	7.776	9.152	10.440	11.147	12.994	< 0.0001
		rSBS	3.973	4.853	5.842	7.457	10.875	
Jejunum	Circular	Control	4.106	5.001	6.131	7.286	8.256	< 0.0001
		rSBS	2.789	2.893	3.605	4.608	5.324	
	Longitudinal	Control	9.238	10.213	10.855	13.489	14.736	< 0.0001
		rSBS	4.830	5.006	6.387	7.278	8.526

Altogether, we can conclude that SBS leads to the nuclei of the cells becoming larger, but the density of the nuclei is lower, which indicates hypertrophy of both the nuclei and the muscle cells themselves.

These results indicate that in the case of SBS, muscle tissue increases significantly in both parts of the small bowel, i.e., jejunum and ileum, which manifests in an increased muscle thickness and an enlarged diameter of the small bowel. So, the leading pathophysiological mechanism of these changes is hypertrophy.

### SBS samples contain more neuronal stem cells in ganglion of myenteric plexus

**Fig. 5:**
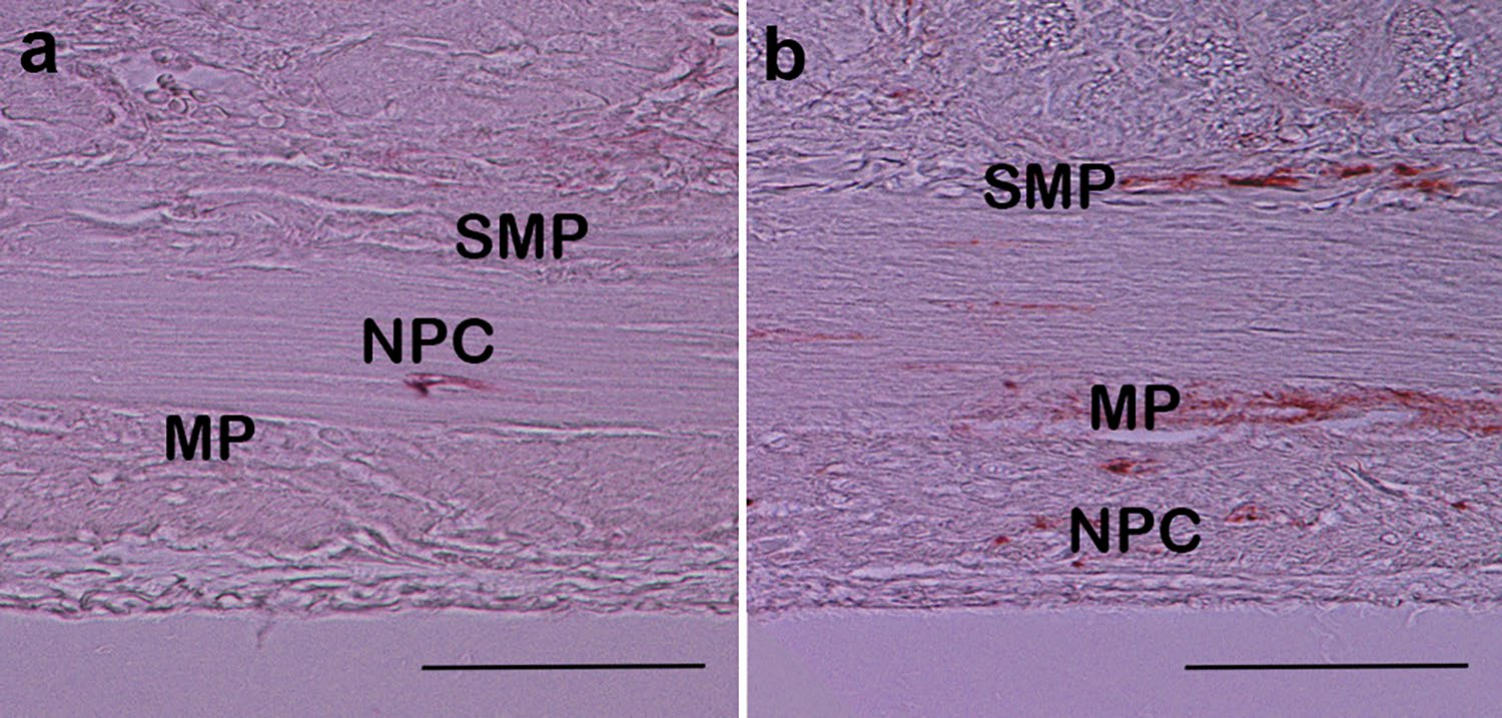
Nestin–peroxidase-stained ganglion of myenteric plexus: **a** control group, **b** short bowel syndrome (rSBS) group (rat). Sections were stained 2 weeks after surgery. MP myenteric plexus, SMP plexus submucosus, NPC nestin-positive cells in muscle layer. In the rSBS group myenteric plexus, plexus submucosa and some cells in the muscle layer are nestin positive (brown color). Scale bar is 50 µm

To explore the role of neurogenesis in intestinal adaptation in SBS, we performed nestin stainings (Fig. 5). Initially, we analyzed the amount of myenteric ganglia expressing nestin in the whole crosscut section (in bowel circumference) to identify whether the amount of myenteric ganglia with upregulation of neuronal stem cells increases in SBS. For these purposes, we used nestin peroxidase staining and quantified the amount nestin-positive myenteric ganglia per section (in bowel circumference).

The analysis revealed that the quantity of nestin-positive myenteric ganglia was significantly increased in the rSBS group compared to control in both parts of the small bowel: in the jejunum (*p* < 0.0001) and ileum (*p* < 0.0001). The number of nestin-positive myenteric ganglia by rSBS group in the jejunum was 8.5 (2–33.25) plexus and in the control group it was was 0 (0–1) plexus; in the ileum by rSBS group it was 5.66 (1–9.25) plexus, by control group it was 0 (0–0.75) plexus.

In SBS, a significantly higher number of ganglia in myenteric plexus with upregulated stem cell population was observed in jejunum and ileum compared to the control. These results suggest that in an effort to adapt to SBS, the myenteric plexus of jejunum and ileum respond with an increase of the number of myenteric ganglia with neuronal stem cells.

**Fig. 6:**
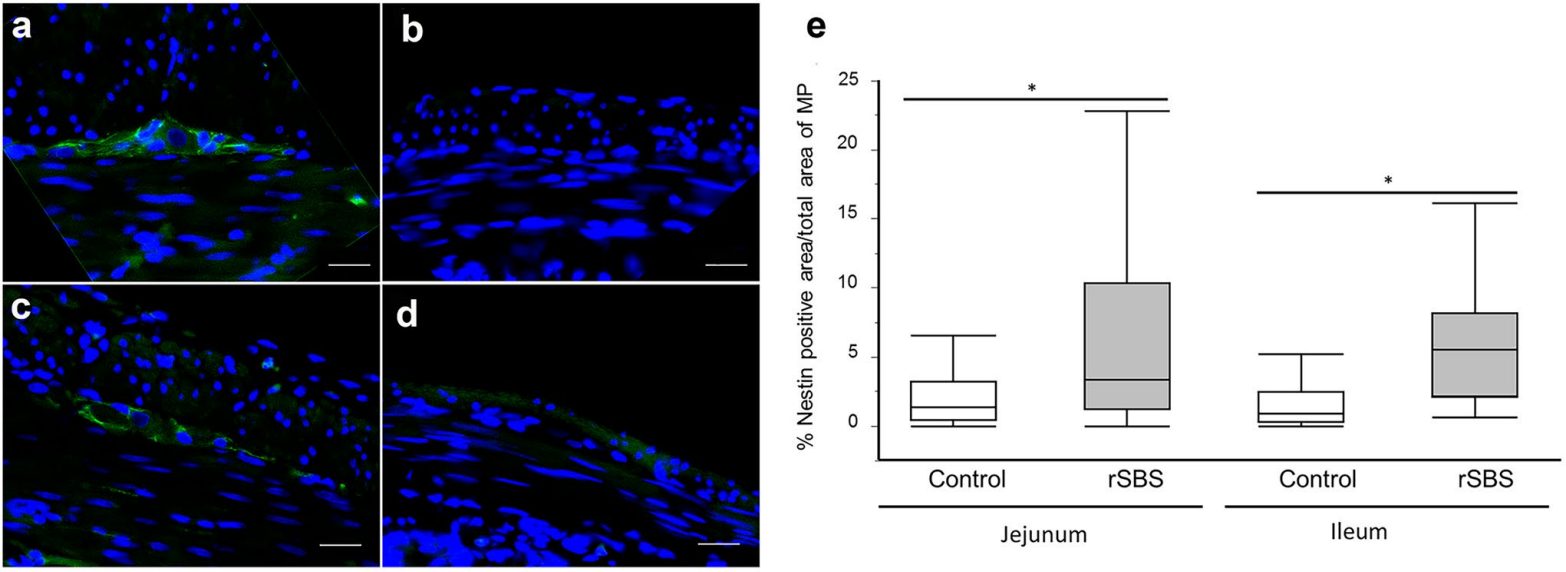
Nestin fluorescence stained ganglion of myenteric plexus of the small intestine. **a** and **c** control group, **b** and **d** short bowel syndrome (rSBS) group (rat). Green color represents nestin fluorescence, **e** ratio of nestin-positive areas within the myenteric plexus compared between control and short bowel syndrome (rSBS). The rSBS small intestine presents more nestin-positive areas within the myenteric plexus. Line within box represents the median; the top and bottom of the box represent the 75th and 25th percentile, respectively. The whiskers indicate the maximum and minimum (not including outliers). Wilcoxon test: *p < 0.0001. Scale bar is 20 µm

Next, we analyzed the expression of nestin in the myenteric gangliaby immunofluorescence (Fig. 6a–d).

Then, we calculated the percentage of nestin-positive area out of the total area of the myenteric ganglia. Statistical analysis showed a significantly larger nestin-positive area in myenteric plexus in the rSBS group compared to controls (jejunum, *p* = 0.0125; ileum, *p* < 0.0001). Specifically, the percentage of nestin-positive area out of the total area of the myenteric plexus in rats with SBS was 3.36% (1.2–10.32%) in the jejunum and 5.51% (2.14–8.17%) and in the ileum; in rats of the control group it was 1.37% (0.42–3.25%) in the jejunum and 0.88% (0.31–2.44%) in the ileum (Fig. 6e).

In SBS, a significantly higher neuronal stem cell population inside the ganglia in myenteric plexus was observed in jejunum and ileum compared to the control. These results suggest that in an effort to adapt to SBS, the myenteric plexus of jejunum and ileum respond with an increase of neuronal stem cells within the ganglia.

Altogether, given that neuronal stem cells are a source of new neurons, our results show that neurogenesis is significantly amplified in the whole small intestine to compensate for SBS.

### There are more nestin-positive areas in intestinal muscle layers of the rats with SBS

Nestin–peroxidase-stained samples (Fig. 5) were analyzed and divided into two groups: (1) samples with a high amount of nestin-positive areas in the muscle layers; (2) samples with a low amount or absent nestin-positive areas in the muscle layers.

In the rSBS group, the ratio of samples with a high amount of nestin-positive areas was 85.7% (jejunum, *p* < 0.05) and 64.3% (ileum, *p* < 0.05), whereas in controls, high amount of nestin-positive areas occurred only in 4.3% (jejunum) and 12.5% (ileum), respectively.

Thus, rSBS presents an accumulation of nestin in the muscle layers.

### Nestin expression correlates with muscle hypertrophy

**Fig. 7:**
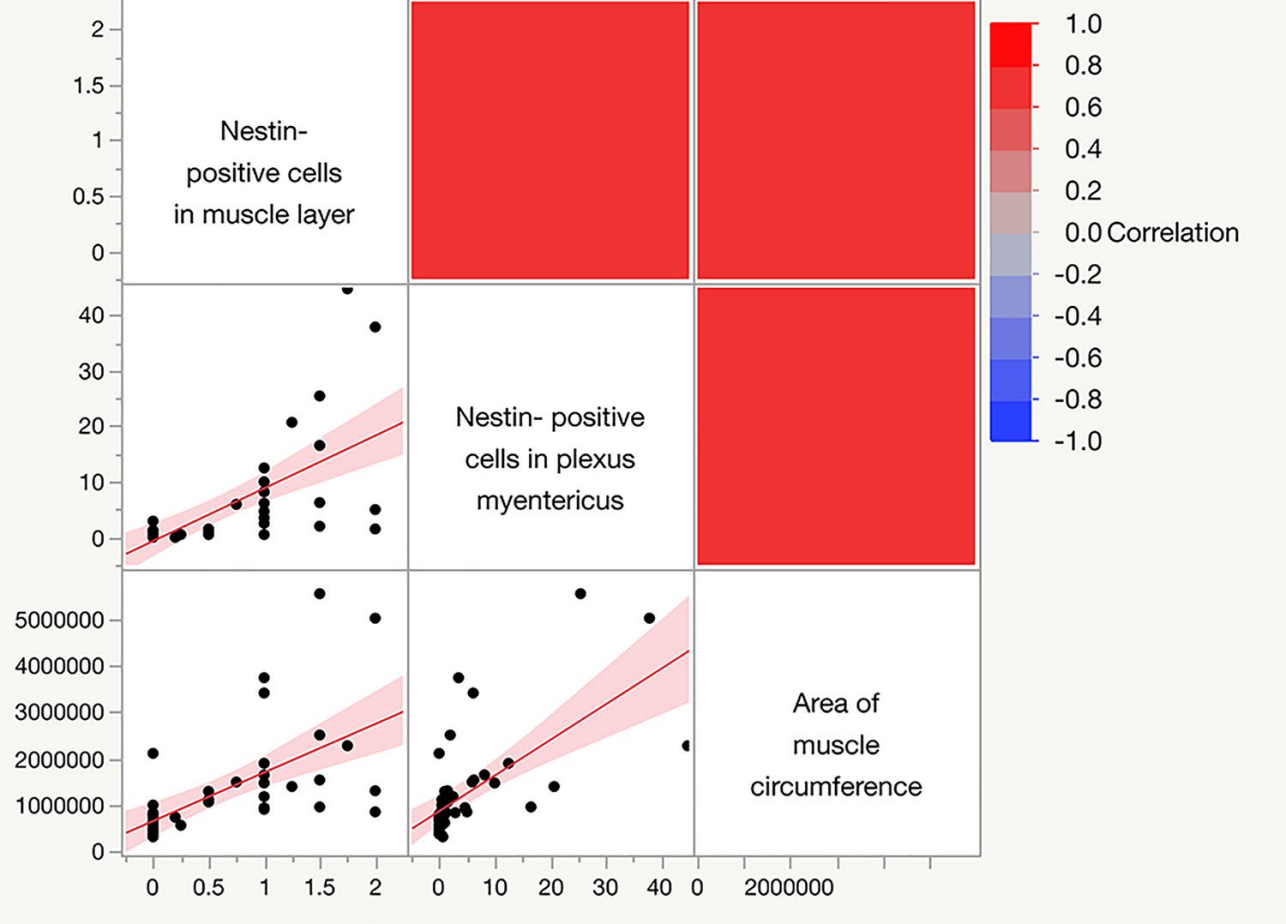
Spearman’s correlation between the area of muscle circumference and the upregulation of nestin expression in the myenteric plexus and in the muscle layer (rat)

We analyzed the correlation between bowel diameter and the thickness, as well as between nestin expression and muscle hypertrophy. A moderate correlation between the bowel diameter and the thickness of the muscle layers (*r* = 0.5447, *p* = 0.0001) was detected with the Spearman’s test. Besides, there was a strong positive correlation between the nestin-positive myenteric plexus and the size of muscle tissue in the muscle layers (the area of muscle circumference) (*r* = 0.7533, *p* < 0.0001) (Fig. 7). Furthermore, the number of nestin-positive cells in muscle layers and the area of muscle circumference showed a significant positive correlation (*r* = 0.7597, *p* < 0.0001) (Fig. 7). The comparison between the quantity of nestin-positive myenteric plexus and the nestin-positive cells in muscle layers also presented a very positive correlation (*r* = 0.8065, *p* < 0.0001) (Fig. 7).

Altogether, there is an association between muscle hypertrophy, the rise of stem cells in muscle layers, and neurogenesis in myenteric plexus in the process of intestinal adaptation to SBS.

### Human data

**Fig. 8:**
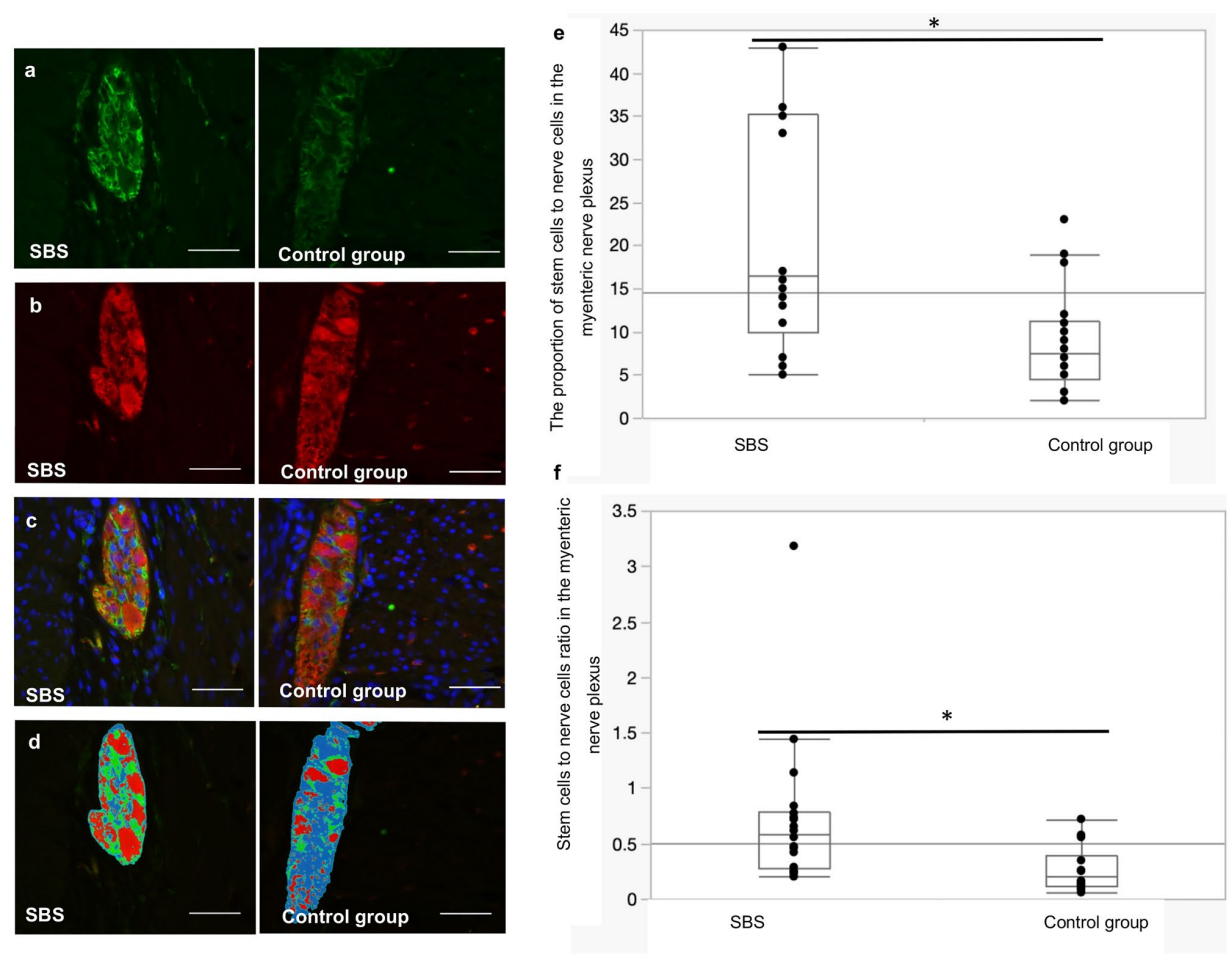
Ganglion of myenteric plexus in a biopsy specimen of the human small intestine. Immunofluorescence staining: stem cells, nestin (green); neurons, PGP 9.5 (red); cell nuclei, DAPI (4′,6-diamidino-2-phenylindole dihydrochloride) (blue). **a** Stem cell research, **b** study of nerve cells, **c** analysis of double staining (stem cells as well as nerve cells), **d** mapping of the myenteric plexus (green—stem cells, red—neurons).** e** The proportion of the area of stem cells to the area of the nerve cells in a cross section of the myenteric plexus.** f** Stem cells to nerve cells ratio in the myenteric plexus Line within box represents the median; the top and bottom of the box represent the 75th and 25th percentile, respectively. The whiskers indicate the maximum and minimum. Wilcoxon test: *p < 0.0001. Scale bar is 50 µm

To investigate whether a similar effect is seen in patients, we examined small bowel tissue samples from patients with and without SBS. The specimens were first stained using HE to identify muscle layers and myenteric plexus and to select the most appropriate sites of the tissue for immunohistochemical staining (Fig. 8). Then, samples were co-stained with antibodies against both PGP 9.5 (a neuronal marker) and nestin (a neuronal precursor marker). This procedure allowed us to observe nerve cells and neuronal stem cells in the samples, to assess the area that these cells occupy in the myenteric plexus (Fig. 8a, b) and to determine the arrangement of these two types of cells (Fig. 8c). Using the microscope software from Keyence BM9000X (Keyence), we mapped the myenteric plexus and quantified the proportion of nestin-positive areas (for neuronal stem cells) and PGP 9.5-positive areas (for neurons) (Fig. 8d).

Results showed a significant increase of neuronal stem cells in patients with SBS in comparison to healthy controls (Wilcoxon test *p* < 0.0001). The observed proportion of stem cells in patients with SBS was 21.3 ± 12.34% of the total area of the myenteric plexus, whereas in the control group it was 6.8 ± 3.98%. Thus, the proportion of stem cells in the myenteric plexus of patients with SBS is at least double the number in the healthy control group (Fig. 8e).

On the other hand, the observed proportion of neuronal cells in the myenteric plexus in patients with SBS did not significantly differ from patients in the control group. However, the ratio of the proportion of stem cells to the proportion of neurons in the myenteric plexus increased significantly (Wilcoxon test *p* < 0.0001). In patients with SBS this indicator was 0.71% ± 0.66% and in the control group it was 0.24 ± 0.16% (Fig. 8f).

## Discussion

During the last few years, changes in muscle layers after massive small bowel resection have been a controversial topic of discussion. While several authors described hypertrophy of intestinal muscle layers as a result of intestinal adaptation in SBS experiments on rats (Chen et al. 2012, 2015, 2013), others did not find evidence for hypertrophy of the muscle layers (Martin et al. 2008).

Despite the fact that the ENS is crucial for bowel motility and its tightly connected to intestinal smooth muscles (Lotfollahzadeh et al. 2021), the changes in ENS after massive small bowel resection have not been published yet.

In this study we have analyzed the intestinal muscle layers and the ENS together as a neuromuscular complex. In order to assess the plasticity of the ENS (Schafer et al. 2009) in the intestinal adaptation to SBS, we used nestin, a well-known ENS precursor cell marker (Vanderwinden et al. 2002; Grundmann et al. 2016; Kulkarni et al. 2017), in both rats and human samples after massive small bowel resection.

Our results show that the thickness of the small bowel muscle wall significantly increased in the SBS group in both proximal and distal segments. Many previous published human data on morphological changes of the small bowel in SBS only focused on the mucosal layer (Tappenden 2014c, b; Doldi 1991; McDuffie et al. 2011; Joly et al. 2009). Besides, while previous studies explored the changes in the muscle layers mainly in the proximal part (in the jejunum, proximal to the resection site) (Martin et al. 2008; Chen et al. 2012, 2015, 2013), here we also analyzed the distal intestinal parts (in the ileum, distal to the resection site), thus we can state that expansion of muscle layer occurs in both jejunum and ileum. We performed smooth muscle actin immunofluorescence staining and Picrosirius Red staining to identify the impact of smooth muscle cell proliferation and increase of connective tissue (collagen fibers) upon changes in the thickness of the muscular layer. Our analysis showed that a considerable increase of smooth muscle cells occurs in SBS and is accompanied by a significant proliferation of connective tissue (collagen). However, the dominating fraction in the muscle layers remains the muscle tissue.

We used the area of muscle circumference as an objective indicator of changes in muscle tissue during intestinal adaptation, because it evaluates both the intestinal diameter and the thickness of the muscular layers. This parameter in the SBS group was significantly increased in both jejunum and ileum compared to the healthy control group. Altogether, we demonstrated that muscle tissue increases significantly in both parts of the small bowel, jejunum and ileum, which results in an increased muscle thickness and an enlarged small bowel diameter.

We also evaluated whether the morphological changes in the muscle layers were a result of hypertrophy or hyperplasia. For that, a morphometric analysis of nuclei (size and density) of the muscle cells was performed. Our data revealed that in SBS, the nuclei of the muscle cells became bigger, but the density of nuclei was reduced, indicating that the leading pathophysiological mechanism of these changes is hypertrophy, which is supported by previous studies (Chen et al. 2015).

In all, our observations indicate that following massive small bowel resection, the intestine responds by developing muscle hypertrophy in an effort to adapt itself to the new situation, where absorptive capacity is strongly reduced.

Concerning the ENS plasticity, we found a higher nestin expression in the myenteric plexus in the remaining small bowel of the SBS samples, which demonstrates that not only the muscle wall but also the ENS responds to bowel resection as part of the adaptation process. These results point to a regenerative potential and plasticity of the ENS in the intestinal adjustment to SBS. Actually, participation of the ENS in the intestinal epithelial growth and repair after SBS has previously been suggested (Toumi et al. 2003; Haxhija et al. 2007).

Nestin expression was also detected in endothelial cells of newly formed blood vessels (Matsuda et al. 2013) and myofibroblasts (Beguin et al. 2012), demonstrating the regenerative character of nestin expression. However, the studies describe that nestin expression in myofibroblasts was markedly lower compared with nestin cells that exhibit a neural stem cell phenotype (Beguin et al. 2012). In our study the nestin expression detected in SBS samples within the muscle layers was comparable to that observed in the myenteric plexus. This could point to an intensified influence of the ENS within the smooth muscle layers and intrinsic activation of neural precursors. It is discussed that after 5-HT_4_ activation, newly born neurons appeared in extraganglionic locations and migrated into myenteric plexus (Liu et al. 2009). In the blood vessels nestin expression was observed only in proliferating endothelial progenitor cells (Suzuki et al. 2010). This showed that the pattern of newly formed microvessels and neural stem cells is highly correlated in all parts of the gut, which illustrates a strong interaction between the enteric nervous and vascular system (Schrenk et al. 2015). That means that nestin proliferation in the muscle layers indicates the proliferation of the ENS. So the higher number of nestin-expressing cells in the muscle layers that we found could suggest that also complementary neuronal progenitor cells outside of myenteric plexus (ganglia) are stimulated during intestinal adaptation. Thus, the ENS may execute a more substantial influence and intrinsic activation of neural precursors on the smooth muscle layers, as was previously suggested (Birbrair et al. 2013). Furthermore, the enteric neurons inside the myenteric plexus (ganglia) and neural precursors on the smooth muscle layers could activate or enhance the proliferation of the muscle cells.

In conclusion, our results show an upregulation of nestin in SBS and evidence that the ENS activates its neurogenic potential and participates in intestinal adaptation.

Moreover, we showed that smooth muscle hypertrophy has a strong positive correlation with the activation of nestin-positive cells in the myenteric plexus (*r* = 0.7533, *p* < 0.0001), as well as in muscle layers (*r* = 0.8065, *p* < 0.0001). A pronounced positive correlation between the proportion of stem cells in the myenteric plexus and smooth muscle hypertrophy allows us to consider that the neuromuscular complex (Barth et al. 2022) of the muscle layers of the small intestine and the ENS plays an essential role in intestinal adaptation by SBS.

In summary, we have identified several readjustments in the gut following SBS, such us an increase in the diameter of the small intestine, hypertrophy of the small intestine muscle layers, and an increase in the proportion of neuronal stem cells in both the myenteric plexus (ganglia) and the small intestine muscle layers. These observations represent the most important pathophysiological mechanisms in SBS and occur in patients with this pathology. Finally, all these changes induce dilated areas of small bowel with muscular wall hypertrophy.

We studied small bowel tissue samples from patients with and without SBS. To explore nerve cells and neuronal stem cells in the samples, double staining with antibodies against both PGP 9.5 (a neuronal marker) and nestin (a neuronal precursor marker) was used. We observed a significant increase of neuronal stem cells in combination with a constant proportion of neurons in the myenteric plexus by SBS, suggesting that the adaptation to SBS manifests also with an expansion of the myenteric plexus.

From a clinical point of view, this phenomenon suggests that the diameter of the small intestine must be measured regularly in patients with SBS. This step will allow the timely recognition of small bowel dilatation and the development of intestinal congestion, as well as enabling future indications for intestinal lengthening surgeries in a prompt manner.

## Conclusion

Smooth muscle hypertrophy, which is accompanied by the proliferation of collagen, and neuroplasticity characterize intestinal adaptation in SBS. Our findings suggest that ENS is tightly connected to changes in intestinal muscle layers and is critically involved in the process of intestinal adaptation to SBS. The ENS activates its neurogenic potential through the neural stem cells and thereby participates in the intestinal adaptation. This process is tightly correlated with an increased expression of neural precursors in the ENS and an increase of the muscle tissue in muscle layers. Since the mechanism of intestinal adaptation is complex and the role of the ENS comprises neural stem cell regulation, further research on enteric glial and neuronal cells is necessary to elucidate the role of enteric neural stem cells in this process.

## Data Availability

The datasets generated and analyzed during the current study are available from the corresponding author on reasonable request.
